# Comparative analysis of the complete mitochondrial genomes of five Achilidae species (Hemiptera: Fulgoroidea) and other Fulgoroidea reveals conserved mitochondrial genome organization

**DOI:** 10.7717/peerj.6659

**Published:** 2019-03-26

**Authors:** Shi-Yan Xu, Jian-Kun Long, Xiang-Sheng Chen

**Affiliations:** 1 Institute of Entomology, Special Key Laboratory for Development and Utilization of Insect Resources of Guizhou, Guizhou University, Guiyang, Guizhou, China; 2 College of Animal Sciences, Guizhou University, Guiyang, Guizhou, China

**Keywords:** Achilidae, Fulgoroidea, Mitochondrial genome, Phylogeny

## Abstract

In the present study, the complete mitochondrial genomes (mitogenomes) of five Achilidae (Hemiptera: Fulgoroidea), *Betatropis formosana*, two new species (*Magadhaideus luodiana* sp. nov and *Peltatavertexalis horizontalis* sp. nov), *Plectoderini* sp. and *Paracatonidia* sp., were sequenced for the first time through next-generation sequencing. The five mitogenomes ranged from 15,214 to 16,216 bp in length, with the typical gene content and arrangement usually observed in Hexapods. The motif “ATGATAA” between *atp8* and *atp6* was found in all the analyzed species. An overlap “AAGCTTA” between *trnW* and *trnC* was observed in the mitogenomes of most Fulgoroidea. The structural and compositional analyses of 26 Fulgoroidea mitogenomes, including the gene rearrangement of five tRNAs (*trnW*, *trnC* and *trnY*; *trnT* and *trnP*), the A + T content and AT-skew of the whole mitogenomes, and the nuclear acid and amino acid compositions of the protein-coding genes (PCGs), revealed family-level differences between Delphacidae and other families (Achilidae, Flatidae, Fulgoridae, Issidae and Ricaniidae). Phylogenetic analyses of 13 protein-coding genes from 26 Fulgoroidea species by maximum likelihood and Bayesian Inference were consistent and well supported the basal position of Delphacidae, a close affinity among the families Flatidae, Issidae and Ricaniidae, and a close relationship between Achilidae and Fulgoridae.

## Introduction

Family Achilidae is a medium-sized group of the superfamily Fulgoroidea (order Hemiptera), called planthoppers. They are worldwide distributed, mainly tropical and sub-tropical regions, and have higher regional endemism than most species in Ricaniidae and Cixiidae of Fulgoroidea, Aphidoidea and Coccoidea of Sternorrhyncha, and Heteroptera (Chen, Yang & Wilson, 1989). Like other hemipteran insects, these phytophagous insects extract plant sap using their sucking and piercing mouthparts, causing abnormal proliferation of plant cells, affecting plant growth and development, spreading plant viral diseases, and leading to severe damage to grain production. Since Stål established the family Achilidae in 1866 (Stål, 1866a, 1866b), a total of 161 genera and nearly 520 species have been so far described all over the world. However, most studies still focus on the identification and description of new species (Hoare, 1934; Linnavuori, 1962; Wilson & Mcpherson, 1980; Emeljanov & Shcherbakov, 2009; Barnard, 2011), a few studies discussed taxonomic relationships among genera or tribes based on morphological characteristics (Metcalf, 1939; O’brien, 1971), and no molecular data was used for the phylogeny of Achilidae.

Insect mitochondrial genome (mitogenome or mtDNA) is generally a highly conserved, double-stranded circular molecule, ranging in length from approximately 14 to 36 kb (Boore, 1999; Cameron, 2014). It contains 37 genes encoding for: 13 protein-coding genes (PCGs: *nad1*-*6* and *nad4l* for NADH dehydrogenase subunits one to six, and 4l; *atp6* and *atp8* for ATP synthase subunits six and eight; *cox1*-*3* for cytochrome c oxidase subunits one to three; *cob* for cytochrome b), 22 transfer RNAs (tRNAs), two ribosomal RNAs (rRNAs: *rrnS* and *rrnL*). In addition, it also includes at least one sequence known as the A + T-rich region of variable length, which plays an important role in the process of transcription initiation and replication regulation (Wolstenholme, 1992; Inohira, Hara & Matsuura, 1997; Osigus et al., 2013). Because of mtDNA’s small size, fast evolutionary rate, relatively conserved gene content and organization, maternal inheritance and limited recombination, mitogenome has been widely used in species identification, population genetics and molecular evolution at various taxonomic levels (Saccone et al., 1999; Zhang et al., 2005; Cao et al., 2006; Ma et al., 2012; Timmermans, Lees & Simonsen, 2014).

In the last 40 years, first-generation sequencing technology, which is also called Sanger sequencing, has been a widely used technology. However, poor quality in the first 15–40 bases of the sequence due to primer binding, deteriorating quality of sequencing traces after 700–900 bases, and the base composition bias (high AT or GC content) and particular structure (poly and stem loop structures) of template DNA fragments have become common challenges of DNA sequencing and main reasons for lengthening research time. In recent years, with the rapid development of sequencing technologies, next-generation sequencing (NGS) technology has become a fast, low-cost and high-throughput method to generate mitogenomic data sets for phylogenetics and other biological questions (Lloyd et al., 2012; Valach et al., 2012; Gillett et al., 2014; Hegedusova et al., 2014; Zhao et al., 2015).

Hemiptera has as many as 92,000 described species and exhibits the highest diversity among hemimetabolous insects (Forero, 2008). Up to date, nearly 75,000 nucleic acid sequences have been published in GenBank, in which the number of complete or nearly complete mitogenomes from Hemipteran insects is only no more than 400. Additionally, the amount of data is Heteroptera > Auchenorrhyncha (Fulgoromorpha and Cicadomorpha) > Sternorrhyncha > Coleorrhyncha, and data distribution is relatively unbalanced: some families do not have sequence information of mitochondrial genomes or even gene fragments. However, only 80 partial mitochondrial sequences (*cox1*, *cob* and *rrnL*) in Achilidae are available, hindering molecular phylogenetic analyses among Achilidae genera and species by using molecular data.

In this study, we sequenced the complete mitogenomes of five Achilidae species, *Betatropis formosana* Matsumura, 1914 (*B. formosana*), *Magadhaideus luodiana* sp. nov (*M. luodiana*), *Peltatavertexalis horizontalis* sp. nov (*P. horizontalis*), *Plectoderini* sp. (*Pl*. sp.) and *Paracatonidia* sp. (*Pa*. sp.), from southwestern China using the NGS technology. In order to reveal the mtDNA molecular features, we also compared Achilidae mitogenomes obtained in the present study with other Fulgoroidea insects. Gene rearrangement, A + T content and AT-skew of 26 whole mitogenomes, and nucleotide and amino acid compositions of 13 PCGs revealed family-level differences between Delphacidae and the other five families (Achilidae, Flatidae, Fulgoridae, Issidae and Ricaniidae). The nucleotide sequences of the 13-PCGs of these five species and 21 other Fulgoroidea insects were used for the maximum likelihood (ML) and Bayesian inference (BI) to investigate the phylogenetic relationships among Achilidae and the other Fulgoroidea subfamilies. Both ML and BI trees well supported the basal position of Delphacidae, a close relationship of Achilidae and Fulgoridae, and an affinity of Ricaniidae, Flatidae and Issidae.

## Materials and Methods

### Species collections and taxonomic identification

Adults of these five Achilidae insects were originally collected from Guizhou and Yunnan province in 2017. All samples were preserved in absolute ethanol immediately and stored at −80 °C in the Institute of Entomology, Guizhou University, Guiyang, China before morphological identification and DNA extraction.

**Figure 1:**
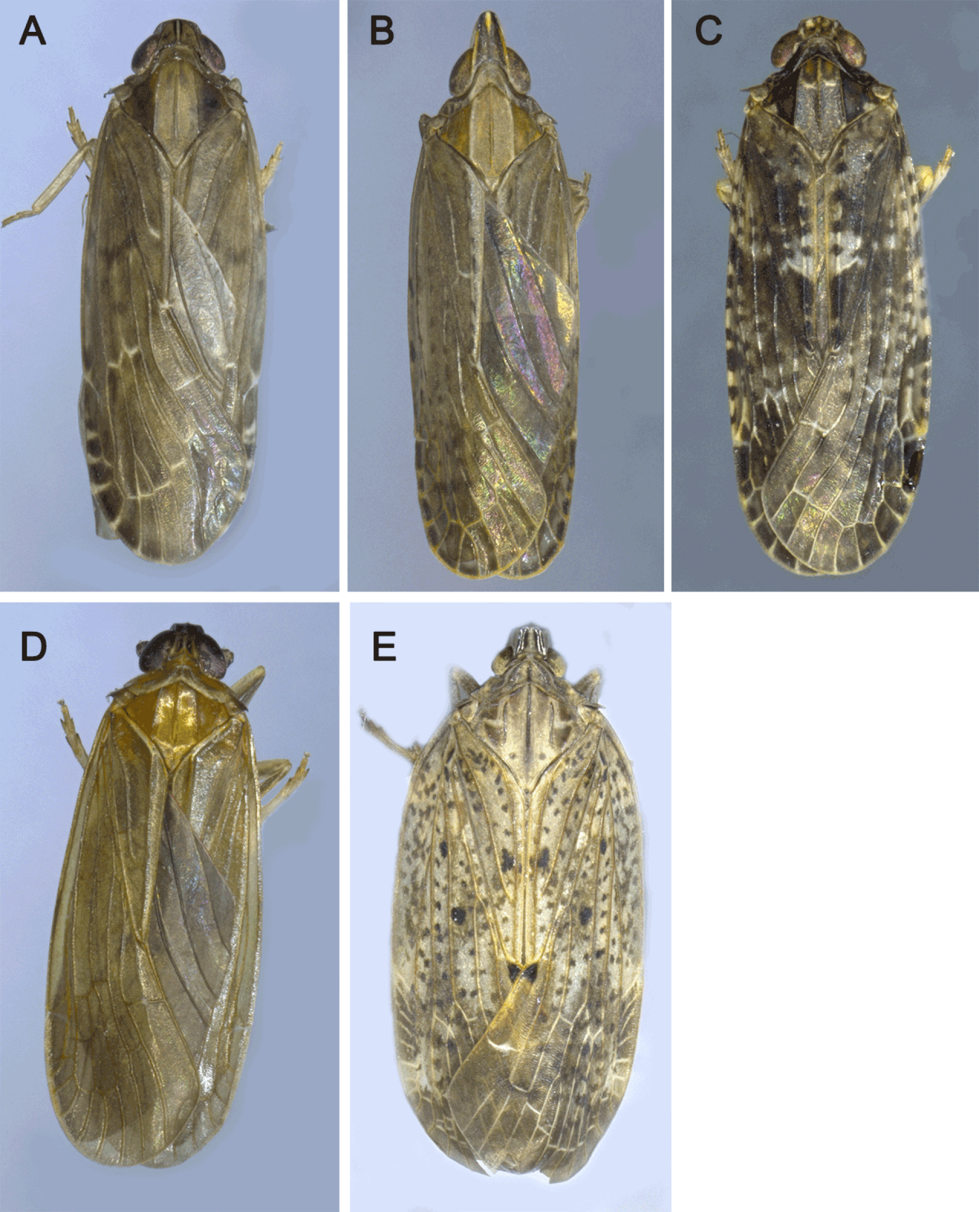
The pictures of five Achilidae insects sequenced in this study. (A) *Plectoderini* sp. (B) *Betatropis formosana* (C) *Magadhaideus luodiana* sp. nov (D) *Peltatavertexalis horizontalis* sp. nov from Luodian, Guizhou province, (E) *Paracatonidia* sp. from Xichou, Yunnan province. Photos of all five insects were taken by Shiyan Xu.

The morphological identification of these five insects were performed by Shiyan Xu. Light microscope images were obtained by a Leica M125 stereomicroscope equipped with a Nikon D7000 digital camera, and were digitally processed with Helicon Focus and Adobe Photoshop CS6 software (Fig. 1). Sex of adult individuals was determined according to structures of the external genitalia. The morphological characteristics for comparison and the data in other species of Achilidae were taken from the previous studies (Chen, Yang & Wilson, 1989; Fennah, 1950, 1956; Long, Yang & Chen, 2015; Long et al., 2017; Xu, Long & Chen, 2018).

### Nomenclatural acts

The electronic version of this article in portable document format will represent a published work according to the International Commission on Zoological Nomenclature (ICZN), and hence the new names contained in the electronic version are effectively published under that Code from the electronic edition alone. This published work and the nomenclatural acts it contains have been registered in ZooBank, the online registration system for the ICZN. The ZooBank Life Science Identifiers (LSIDs) can be resolved and the associated information viewed through any standard web browser by appending the LSID to the prefix http://zoobank.org/. The LSID for this publication is: urn:lsid:zoobank.org:pub:D1E6581B-15A0-4277-B00E-0564ABD04DE1. The online version of this work is archived and available from the following digital repositories: PeerJ, PubMed Central and CLOCKSS.

### DNA extraction and sequencing

Total genomic DNA was extracted from each Achilidae species with TIANGEN Genomic DNA Extraction Kit (TIANGE, Beijing, China) according to the manufacturer’s instructions. Quality of the extracted DNA was checked on 1% agarose gel, sheared to 250–300 bp segments, A- and B- tailed and ligated to Illumina paired-end (PE) adapters. About 350 bp ligated fragments were quality selected on agarose gel and amplified to yield the corresponding short-insert libraries. Subsequently, HiSeq X 10 was used to sequence PE reads and the length of each read was 150 bp. Raw data was adapter clipped and qualified using tools fastx clipper and fastq_quality_filter in FASTAX-Toolkit (http://hannonlab.cshl.edu/fastx_toolkit/index.html), respectively. The clean data was then used for mitochondrial genome reconstruction by SOAPdenovo (Luo et al., 2012) and GapFiller (Boetzer & Pirovano, 2012) with default parameters under the references of previously published mitochondrial genomes of related species. Finally, we gained complete mitogenomes of these five insects. All the detailed information is shown in Table S1.

### Genome annotation and analysis

The initial annotation of the five mitogenomes, including gene prediction and non-coding RNA, were conducted using MITOS WebServer (http://mitos.bioinf.uni-leipzig.de/index.py) (Bernt et al., 2013). PCGs were translated into putative proteins on the base of the invertebrate genetic code using MEGA v5.1 (Tamura et al., 2011).

The identification and structure prediction of tRNAs were performed using MITOS and tRNAScan-SE Search Server available online (http://lowelab.ucsc.edu/tRNAscan-SE) with invertebrate codon predictors (Lowe & Eddy, 1997). While the undefined tRNAs were further compared through alignments with the nucleotide sequences of other species (Negrisolo, Babbucci & Patarnello, 2011; Zhu et al., 2017). The predicted secondary structures of all tRNAs were drawn by Adobe Illustrator CS6. Tandem repeats were identified by the tandem repeats finder online server (Benson, 1999).

The base composition of nucleotide sequences was described by skewness and was measured according to the following formulas (Perna & Kocher, 1995): AT-skew = (A − T)/(A + T) and GC-skew = (G − C)/(G + C). The values of nucleotide composition and relative synonymous codon usage (RSCU) were calculated using MEGA v7.0.26 (Tamura et al., 2011; Liu et al., 2014). The sequence data of the five insect mitogenomes have been deposited to GenBank under the accession numbers MH324927–MH324931 for *Betatropis formosana* (*B. formosana*), *Magadhaideus luodiana* sp. nov (*M. luodiana*), *Peltatavertexalis horizontalis* sp. nov (*P. horizontalis*), *Plectoderini* sp. (*Pl*. sp.) and *Paracatonidia* sp. (*Pa*. sp.), respectively.

### Phylogenetic analyses

Besides five newly sequenced mitogenomes, 21 complete or nearly complete mitogenomes from Fulgoroidea insect species were used in phylogenetic analysis, with *Pomponia linearis* and *Meimuna opalifera* in Cicadoidea, and *Cosmoscarta* sp. and *Aeneolamia contigua* in Cercopoidea as outgroups (Liu et al., 2014; Song, Cai & Li, 2017). Their accession numbers and information are listed in Table S2. The nucleotide sequences of all PCGs were aligned based on amino acid sequences with Muscle criterion (Edgar, 2004) implemented in MEGA v7.0.26, respectively. The subsequent alignments were then concatenated by Bioedit v7.0.5.3 (Hall, 1999) and were used to reconstruct phylogenetic trees by ML and BI, respectively. The best substitution models and partition schemes for both ML and BI trees (Table S3) were estimated using PartitionFinder v2.1.1 (Lanfear et al., 2017), with the greedy algorithm. For ML analysis, IQ-Tree v1.4.3 (Nguyen et al., 2015) was used with 1,000 replicates of ultrafast likelihood bootstrap (Minh, Nquyen & Von Haeseler, 2013) to obtain bootstrap branch support values. Bayesian analysis was conducted with MrBayes on XSEDE v3.2.6 (Huelsenbeck & Ronquist, 2001) available from the CIPRES Science Gateway (https://www.phylo.org/) (Miller, Pfeiffer & Schwartz, 2010). Four simultaneous Markov chains ran for 20 million generations and sampled every 1,000 generations after discarding the first 25% “burn-in” trees. Nodal supports were assessed by the value of Bayesian posterior probabilities (BPP). The consensus trees were viewed and edited by Figtree v1.4.3.

## Results

### Genome structure, organization and nucleotide composition

**Figure 2:**
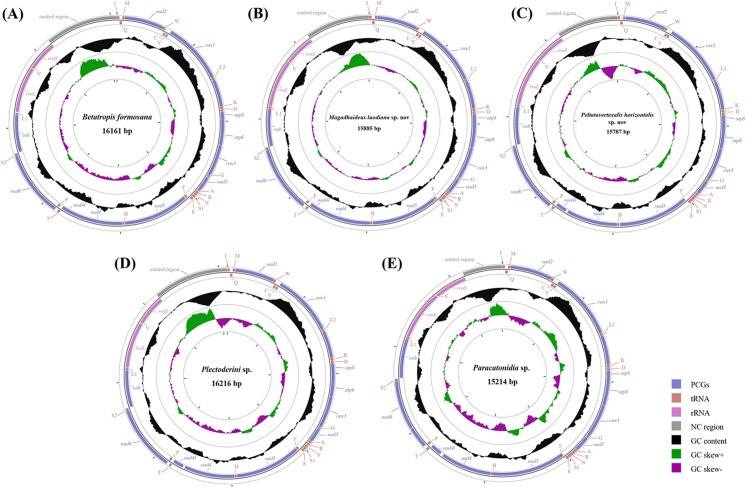
Circular diagram of five Achilidae mitochondrial genomes. (A) *Betatropis formosana* (B) *Magadhaideus luodiana* sp. nov (C) *Peltatavertexalis horizontalis* sp. nov (D) *Plectoderini* sp. (E) *Paracatonidia* sp. The genes outside the map are coded on the heavy strand (H-strand), whereas the genes on the inside of the map are coded on the light strand (L-strand). The middle circle (black) displays the GC content and the paracentral circle (purple & green) displays the GC skew. Both GC content and GC skew are plotted as the deviation from the average value of the total sequence. The PCGs and rRNAs are the standard abbreviations. Each tRNA is denoted as a one-letter symbol according to the IUPAC-IUB single-letter amino acid codes.

**Figure 3:**
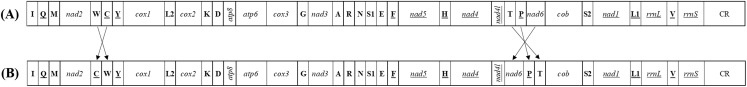
The mitochondrial gene arrangements detected from (A) Achilidae, Flatidae, Fulgoridae, Flatidae, Issidae and Ricaniidae and (the putative ancestral arthropod) and (B) Delphacidae. Arrows indicate the rearrangements of genes or gene clusters.

Each of all five newly sequenced mitogenomes was a circular double-stranded DNA molecule (Fig. 2), containing 37 genes (13 PCGs, two rRNAs and 22 tRNAs) and a non-coding control region, as commonly found among metazoans (Negrisolo, Babbucci & Patarnello, 2011; Liu et al., 2014; Wang & Tang, 2017). There were no differences in the gene order of all the five mitogenomes compared with the putative ancestral gene order (Table S4) (Dai et al., 2014, 2016). However, the five tRNA genes (*trnW*, *trnC* and *trnY*; *trnT* and *trnP*) order of Delphacidae mitogenomes differed from the putative ancestral gene order observed in Achilidae, Flatidae, Fulgoridae, Issidae and Ricaniidae mitogenomes (Fig. 3). In contrast to the traditional tRNA genes order, *trnC* and *trnW* have exchanged position reciprocally. A similar location exchange was also observed between *trnT* and *trnP*, and the location of them and *nad6* also changed. A total of 23 genes were located on the heavy-strand (H-strand) with the remaining 14 genes on the light-strand (L-strand).

The sizes of the five Achilidae mitogenomes sequenced in this study ranged from 15,214 (*Pa*. sp.) to 16,216 bp (*Pl*. sp.) (Table S4), of which the length differences varied from 55 bp between *Pl*. sp. and *B. formosana* to 1,002 bp between *Pl*. sp. and *Pa*. sp. The differences were congruent with those of control regions.

**Figure 4:**
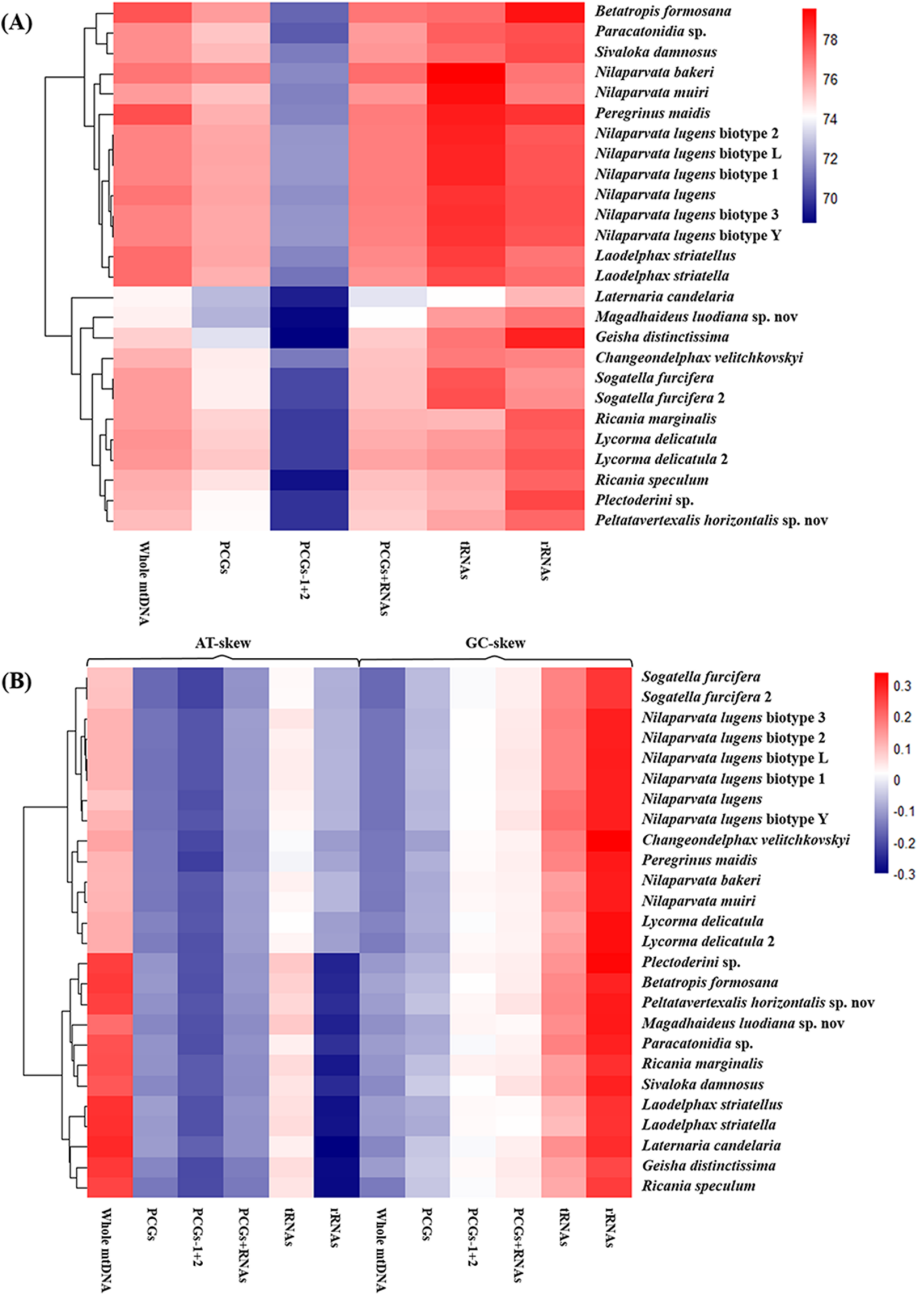
Nucleotide composition of various datasets of Fulgoroidea mitogenomes. Hierarchical clustering of Fulgoroidea species (*y*-axis) based on (A) the A + T content; (B) the AT-skew and GC-skew.

The nucleotide composition of 26 complete or nearly complete mitogenomes in Fulgoroidea was investigated through the calculation of A + T content, AT-skew and GC-skew in percentages. The variation of AT% ranged from 74.3% to 77.8%, with the average value equal to 76.32% (Fig. 4A; Table S5). The overall A + T content of five newly sequenced mitogenomes ranged from 74.4% in *M. luodiana* to 77.7% in *B. formosana*. The high AT-skew values among analyzed mitogenomes (0.091–0.284) indicated the occurrence of more As than Ts, which was also observed in other examined Fulgoroidea mitogenomes (Fig. 4B; Table S5). Moreover, values of the AT-skew of these five mitogenomes were higher than those of Delphacidae insects, which was mainly due to a relatively high AT skewness of PCGs at all codon positions.

### Protein-coding genes

Six kinds of triplet initiation codons (ATN, GTG and TTG) have been frequently reported in the mitogenomes of other insects. In the five newly sequenced mitogenomes, except for *nad1* of *Pl*. sp., *M. luodiana* and *Pa*. sp. started with GTG and *nad5* of *M. luodiana* started with GTG, the typical ATN codons were used as a start condon. The complete stop codon TAA was more frequently used than TAG, while incomplete termination codon TA/T was found in PCGs of five mitogenomes. This latter evidence well documented in other insect mitogenomes and with functionality of stop codons supposed to be restored through post-transcriptional polyadenylation (Boore, 2004; Cao et al., 2006; Ojala, Montoya & Attardi, 1981).

**Figure 5:**
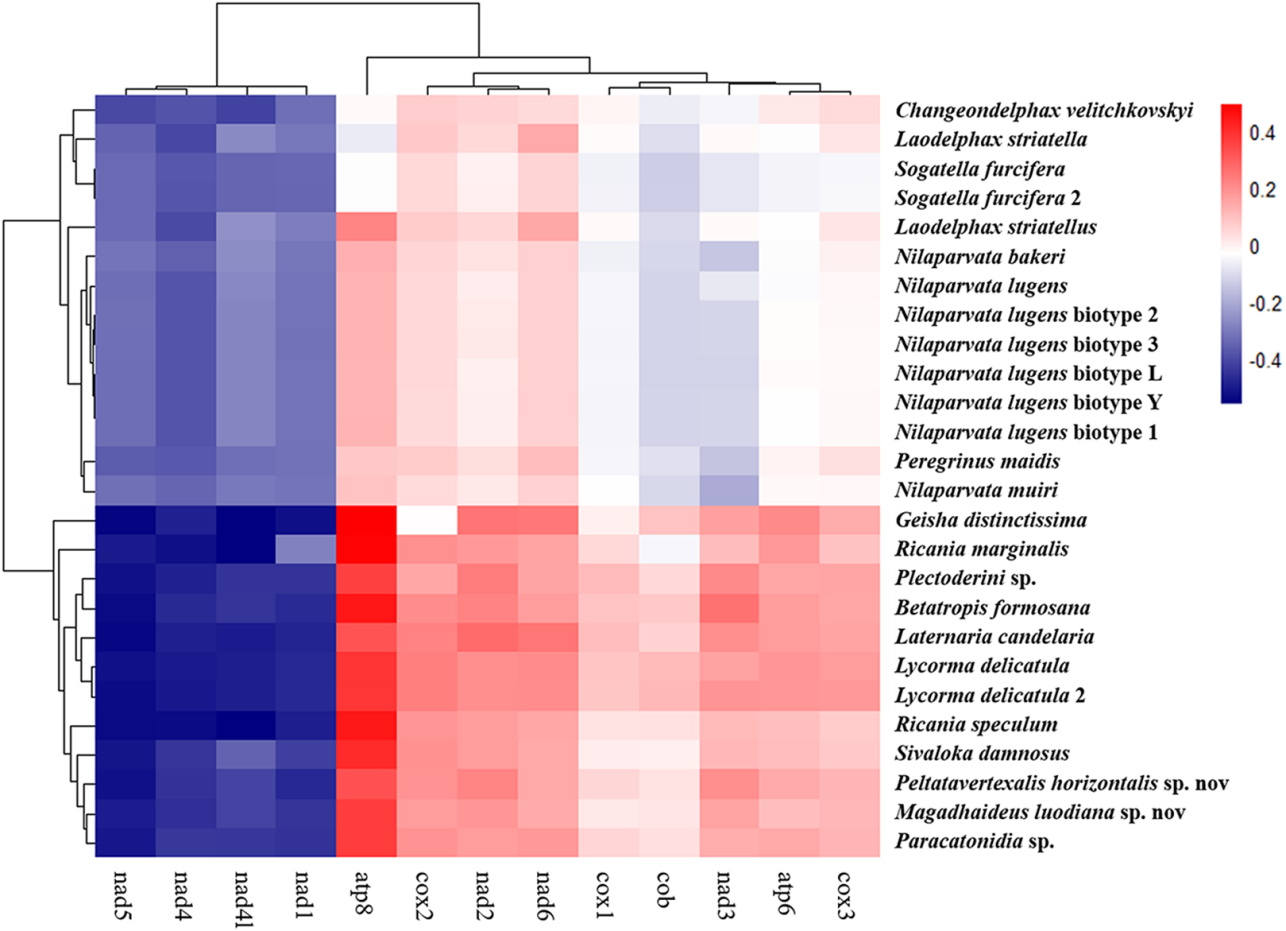
AT-skew of the PCGs of Fulgoroidea mitogenomes.

**Figure 6:**
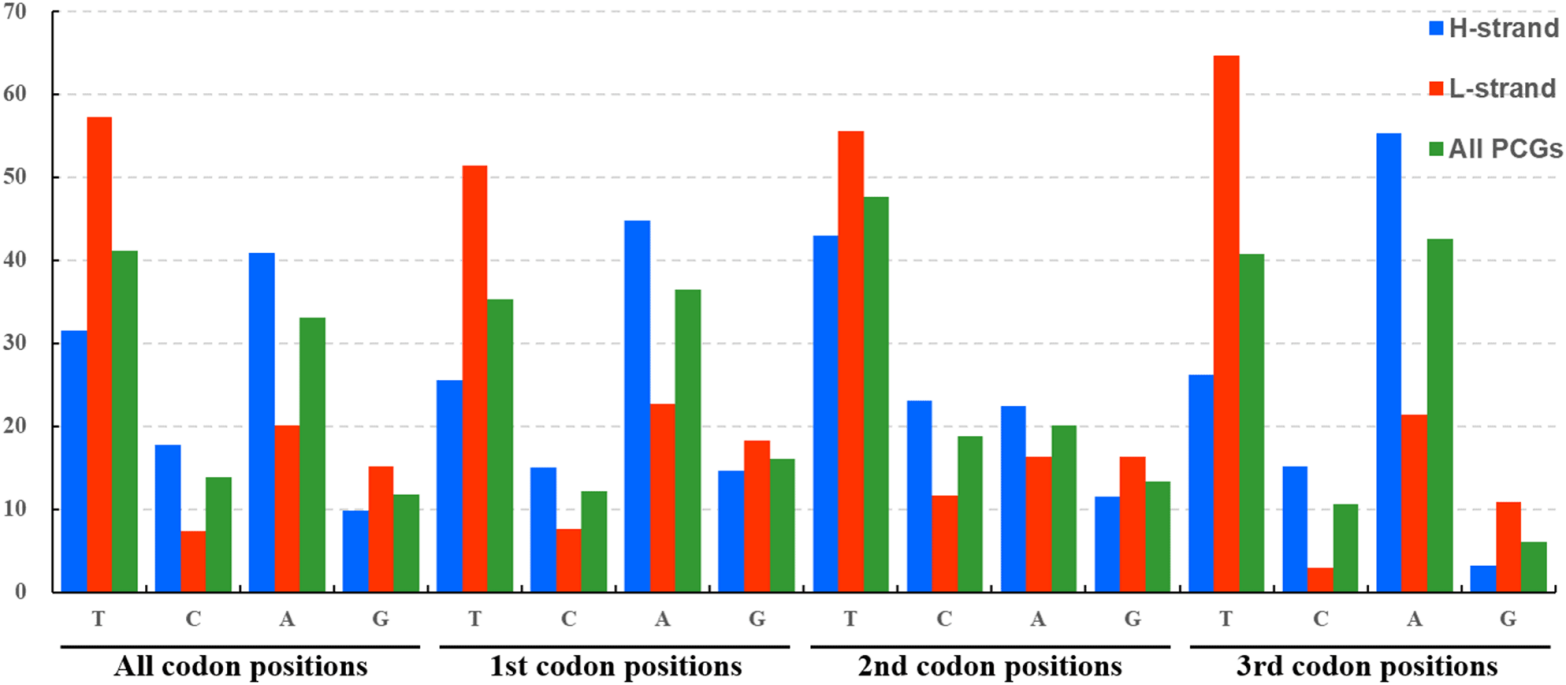
Nucleotide compositions of three codon positions of the PCGs encoded in H- and L-strand.

The average nucleotide composition analysis of the PCGs of Fulgoroidea mitogenomes showed that the PCGs in the H-strand had much higher AT-skew than those in the L-strand (Fig. 5). Moreover, the AT-usage differences between both strands were more significant in Achilidae, Fulgoridae, Flatidae, Issidae and Ricaniidae than in Delphacidae. The nucleotide composition of the PCGs of the five mitogenomes showed an extreme AT nucleotide bias at all codon positions (Fig. 6). Furthermore, the average nucleotide composition of the first and third codon positions of the H-strand contained more A than T, while the second had more T than A. However, in the L-strand, all the three positions had more T than A.

**Figure 7:**
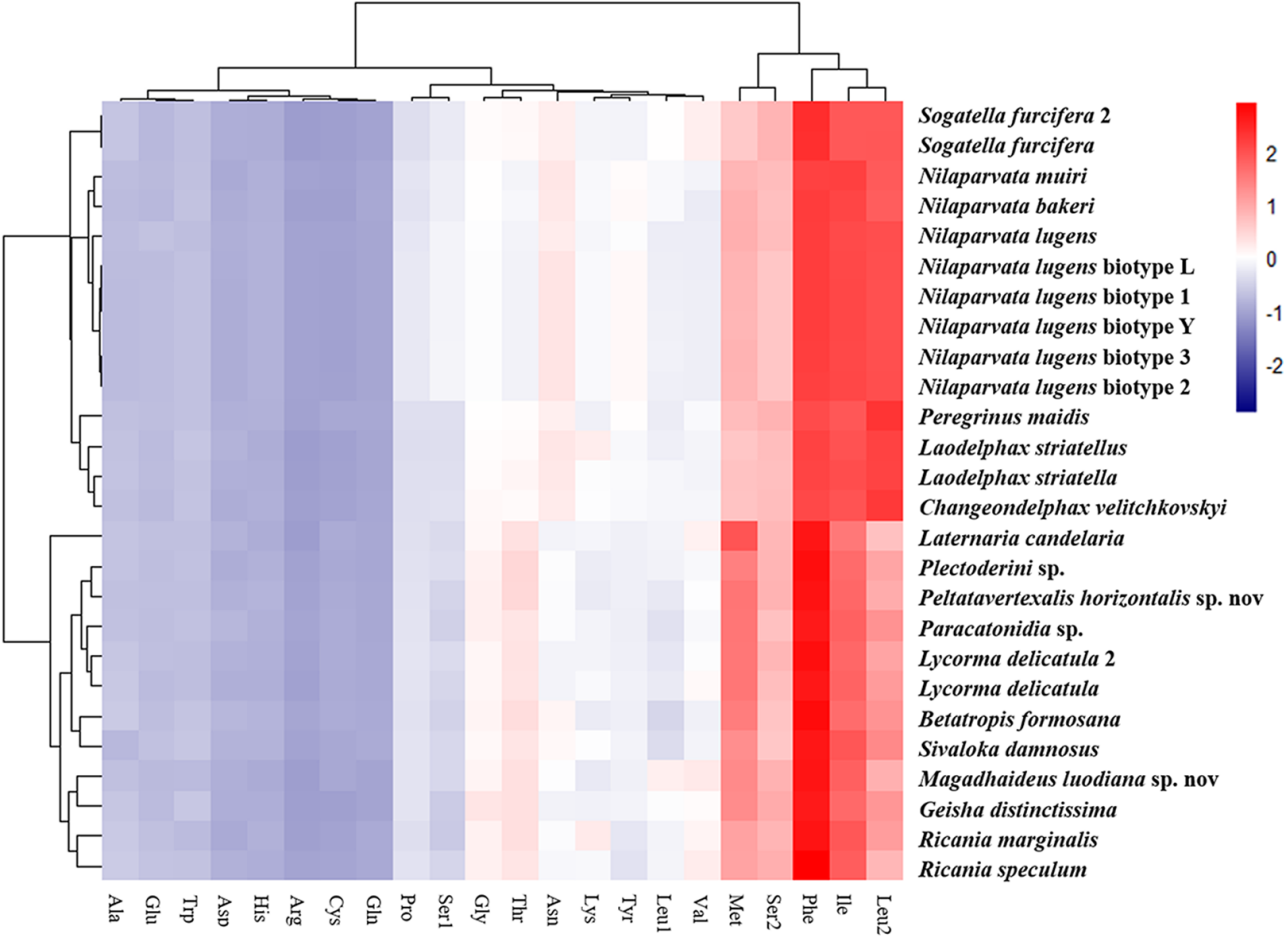
The amino acid composition of 13 PCGs of the Fulgoroidea mitogenomes.

The amino acid composition analysis of the proteins (Fig. 7; Table S6) coded by the mitogenomes also showed that Phe, Ile, Met, Leu2 and Ser2 were the five most common amino acids, making up more than half of the amino acids in sum (Table S6). The same features were also found in the mitogenomes of five Callitettixini species (Hemiptera: Cercopidae) (Liu et al., 2014), while other analyses based on mitogenomes showed significant differences (Wang & Tang, 2017; Yu et al., 2017). Additionally, there were more Phe and Met but less Ile and Leu2 in Achilidae, Fulgoridae, Flatidae, Issidae and Ricaniidae than in Delphacidae. In other words, the amino acid composition of the analyzed mitogenomes have family-level characteristics (CDspT, codons per thousand codons: Phe > Ile > Met > Leu2 > Ser2 in Achilidae, Flatidae and Fulgoridae; Phe > Ile > Leu2 > Met > Ser2 in Ricaniidae and Issidae; Phe > Ile/Leu2 > Met/Ser2 in Delphacidae).

Theoretically, 20 amino acids in the mitochondria corresponding to 22 tRNAs and 62 codons, indicated that each tRNA must recognize at least two types of codon. However, RSCU indicated strong biases of codon usage and amino acid composition (Fig. S1; Table S7). The codon usage comparison of amino acids Leu indicated that the codon UUN was more frequently used than the codon CUN. Some codons (GCG, GGC, GTG and CGC) were rarely used in the PCGs, which was also found in other insects (Chai, Du & Zhai, 2012; Sun et al., 2009; Wang & Tang, 2017).

### tRNAs and rRNAs

**Figure 8:**
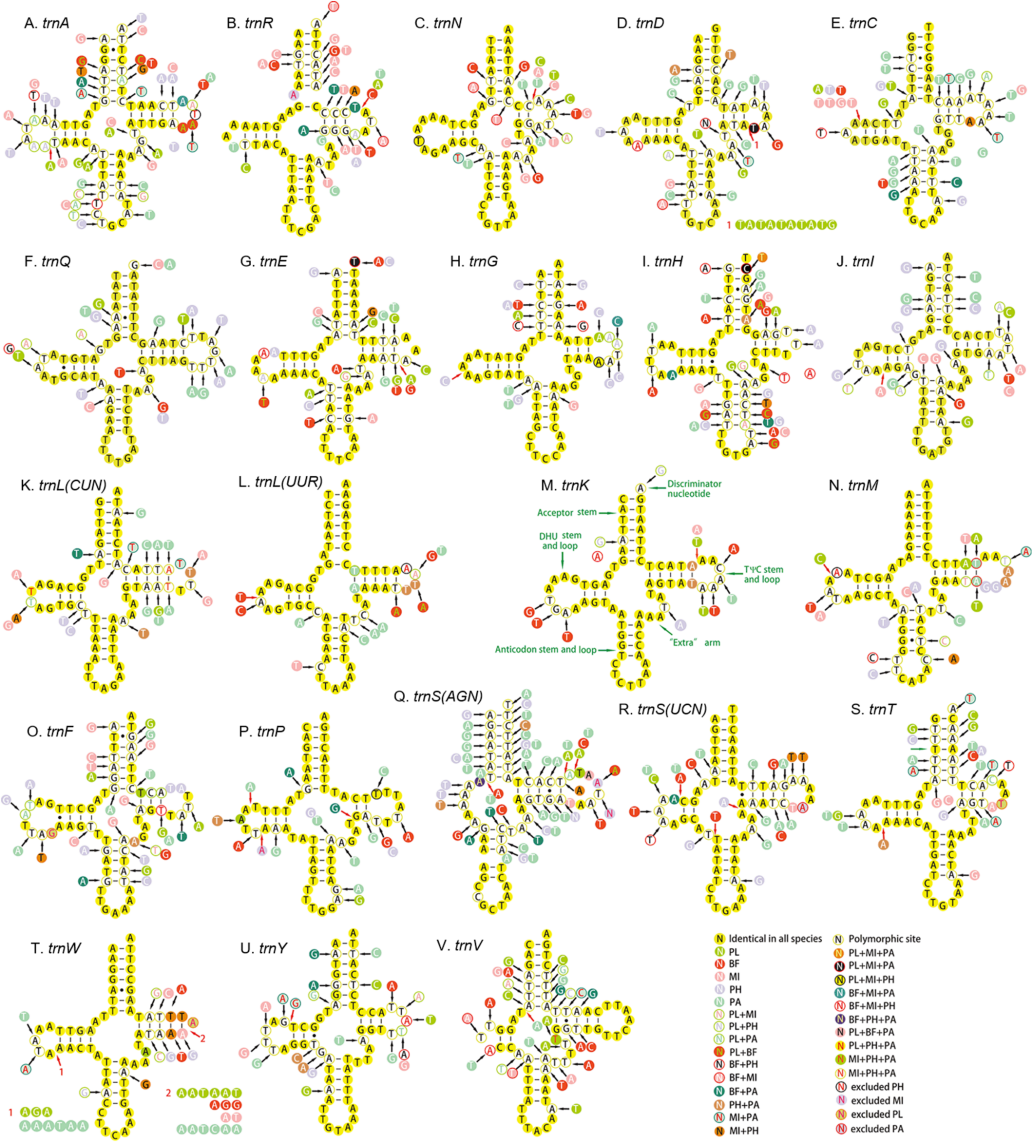
Secondary structure of tRNA families in five Achilidae mitogenomes. (A) *trnA*; (B) *trnR*; (C) *trnN*; (D) *trnD*; (E) *trnC*; (F) *trnQ*; (G) *trnE*; (H) *trnG*; (I) *trnH*; (J) *trnI*; (K) *trnL(CUN)*; (L) *trnL(UUR)*; (M) *trnK*; (N) *trnM*; (O) *trnF*; (P) *trnP*; (Q) *trnS(AGN)*; (R) *trnS(UCN)*; (S) *trnT*; (T) *trnW*; (U) *trnY*; (V) *trnV*. PL, *Plectoderini* sp; BF, *Betatropis formosana*; MI, *Magadhaideus luodiana* sp. nov; PH, *Peltatavertexalis horizontalis* sp. nov; PA, *Paracatonidia* sp.

All the 22 tRNAs of the five mitogenomes were identified with the total length between 1,397 and 1,417 bp. A total of 14 tRNAs were encoded by the H-strand and the other (eight) by the L-strand. The length of tRNAs ranged from 55 to 72 bp. Except for the *trnS1*, which lacked dihydrouridine (DHU) arm and formed a simple loop, all the tRNAs could be folded into canonical clover-leaf secondary structure with an aminoacyl arm of seven base pairs and anticodon arm of five base pairs (Fig. 8). Moreover, an unpaired A nucleotide was found in the anticodon stem of *trnS1*, and similar pattern was also detected in the acceptor stem of *trnR*. The unusual phenomenon had been reported in other hemipterans (Li et al., 2016; Su et al., 2018; Yuan et al., 2015). The Watson–Crick base pairs (A–T and G–C) were frequently observed in stem regions, while G–U wobble and mismatched pairs can also be found.

The percentage of identical nucleotides (%INUC) in the five mtDNAs was calculated (Table S8). The *trnK* had the highest %INUC (81.7%), and four tRNAs (*trnL2*, *trnG*, *trnM* and *trnN*) on the H-strand and three tRNAs (*trnP*, *trnQ* and *trnL1*) on the L-strand showed a higher %INUC (>70%) in five mitogenomes, while the %INUC of *trnS1* located on the H-strand was only 25%. Moreover, in these conserved regions, six and five G–U wobble base pairs were observed in acceptor and DHU arm, respectively; while three (one AC in *trnL2*, one GA in *trnW* and one AA in *trnK*) mismatched base pairs were found. The above results indicated a high-level conservation of tRNAs encoded on the H-strand.

The large ribosomal RNA (*rrnL*) and small ribosomal RNA (*rrnS*) genes were oriented on the L-strand of the five mitogenomes, with a high A + T content (>70%), a negative AT-skew and a positive GC-skew (Table S5).

### Gene overlaps and non-coding A + T-rich Region

**Figure 9:**
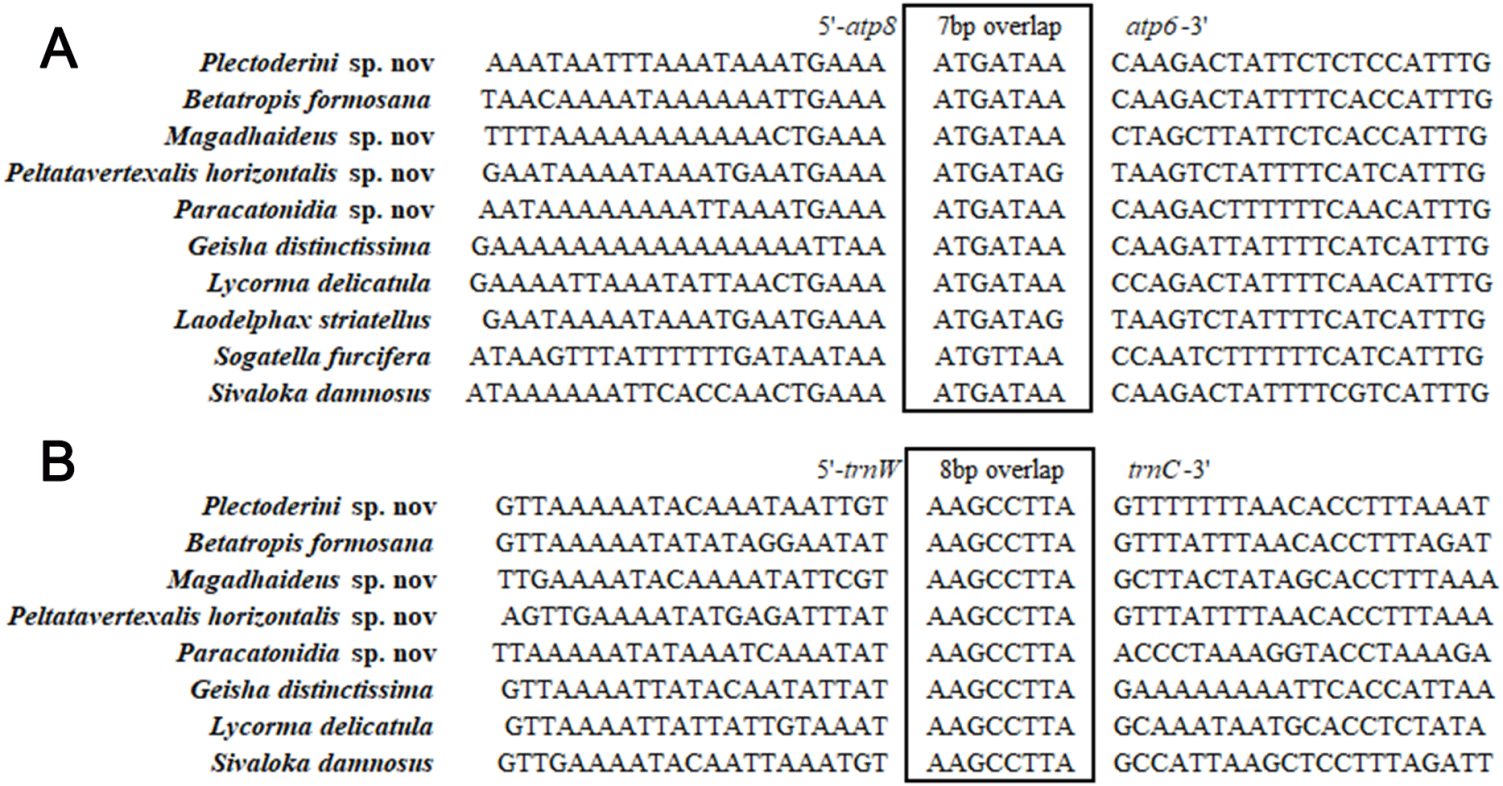
Sequence alignments of (A) *atp8*/*atp6* and (B) *trnW*/*trnC* of insects in Fulgoroidea. The boxed nucleotides indicate the seven and eight bp conserved overlaps, respectively.

Different from the nuclear genome, there are short spaces or even overlaps between genes in mitochondrial genomes. The total length of overlapping regions in the five mitogenomes ranged from 42 bp in *Pl*. sp. mitogenome to 52 bp in the mitogenome of *Pa*. sp. The longest overlaps (20 bp) occurred between *trnF* and *nad5* in *Pa*. sp. mitogenome. A seven-bp overlap (ATGATAA) between *atp8* and *atp6* found in other insect mitogenomes was also observed in the mitogenomes analyzed in this study (Fig. 9A). This fragment was thought to be translated as a bicstron (Stewart & Beckenbach, 2005). However, another seven-bp overlap between *nad4l* and *nad4* generally found in other insect mitogenomes was not observed in this study. An eight-bp overlap (AAGCCTTA) between *trnW* and *trnC* was detected in five newly sequenced mitogenomes, which was absent in species of Delphacidae (Fig. 9B). In the mitogenomes of *Pa*. sp., the total lengths of intergenic spacers (114 bp) was much longer than those in the other four Achilidae species (Table S4).

Compared with the other regions, the A + T-rich regions of the five mitogenomes between the *rrnS* and *trnM* exhibited more variation in length, ranging from 908 bp in *Pa*. sp. to 1,850 bp in *Pl*. sp. (Table S4). It is obvious that the length differences among them were consistent with the total length differences of their mitogenomes. This region also harbored the highest A + T content (avg. 82.91%) in the mitogenomes, while AT skewness and GC skewness were significantly different, which was also observed in other insect mitogenomes from Fulgoroidea (Table S5).

Two typical repeating sequences scattered throughout the five entire mitogenomes, were identified. Two repeat sequences (135 × 5 and 21 × 13) were found in the A + T-rich region of *Pl*. sp. mitogenome, comprising half of the whole control region (Table S9). However, the lengths of the repeat sequences in other control regions decreased with the length of corresponding control regions. This feature indicated that characteristics of these regions in Achilidae were taxon-specific, and the different sizes or copy numbers of repeat units had some influences on the size of the control regions and also of the mitogenomes.

### Phylogenetic analyses

**Figure 10:**
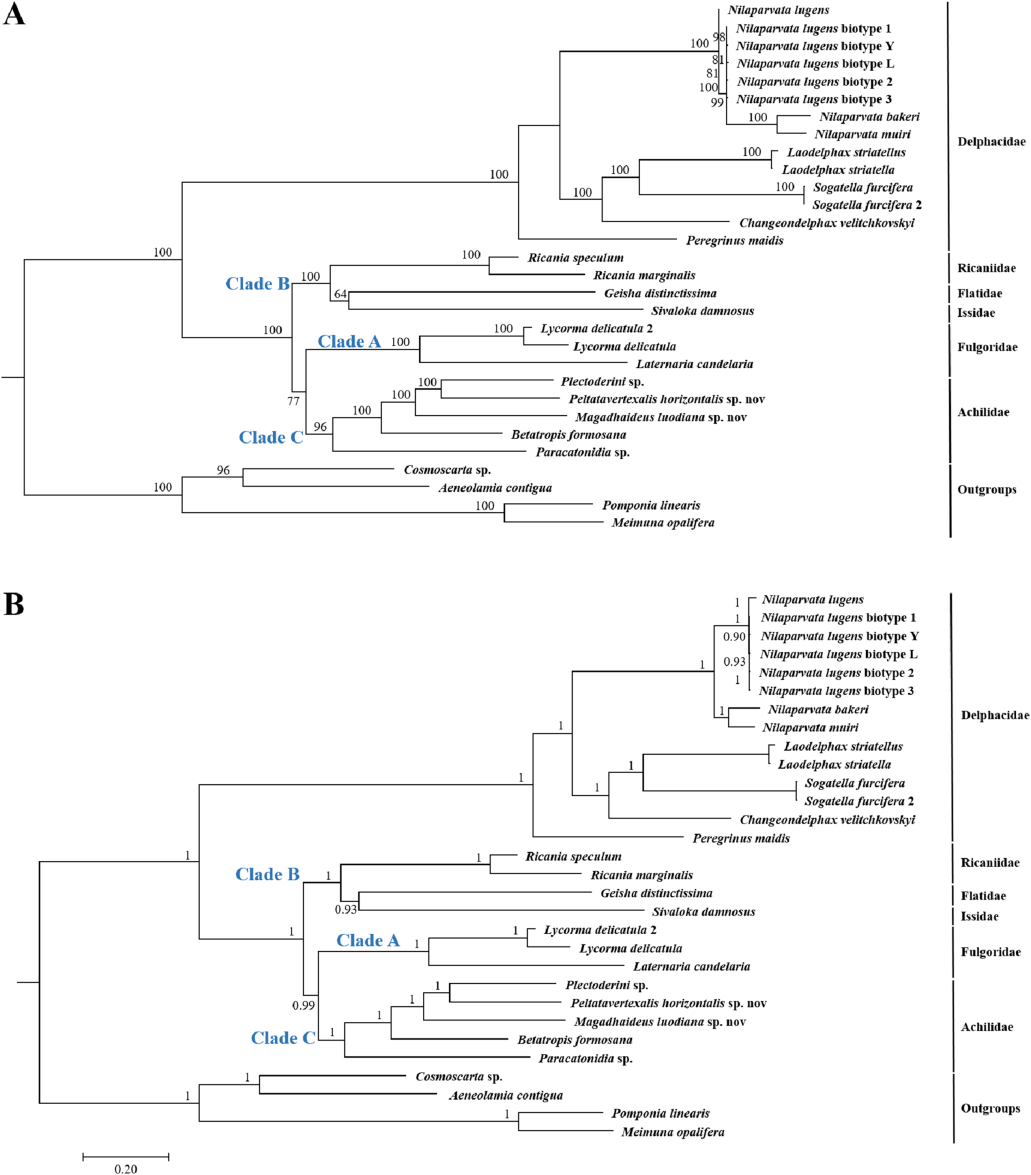
The (A) ML and (B) BI phylogenetic trees based on the nucleotide datasets for 13 PCGs from the mitochondrial genomes of 30 species. All the probability values and bootstrap values of the branches were indicated, respectively.

Because of limited mitogenome sequences of Fulgoroidea, we included in the phylogenetic analyses only 26 species to understand the evolutionary relationships of Achilidae with the other Fulgoroidea families. Phylogenetic trees of ML and BI analyses were constructed based on 13 PCGs nucleotides sequences from 30 species (Table S2; Fig. 10). Overall, both trees generated the same topology, and BI analysis provided more resolution with strong supports than ML analysis. In both trees, Fulgoroidea is divided into two groups: Delphacidae and ((Ricaniidae, (Flatidae, Issidae)), (Fulgoridae, Achilidae)). Four families (Achilidae, Delphacidae, Fulgoridae and Ricaniidae) including more than two species formed separate branches in both BI and ML analyses (bootstrap support values (BS) ≥ 96, and BPP = 1.00).

In both ML and BI topologies, the basal position of Delphacidae was well supported with high node values (BPP/BS = 1/100), which was also supported by many previous studies based on morphological characters and molecular data (Muir, 1930; Asche, 1987; Bourgoin, Steffen-Campbell & Campbell, 1997; Yeh, Yang & Hui, 2005; Gnezdilov, 2016). In both topologies, relationships between Achilidae (Clade C) or Fulgoridae (Clade A) and other families (Clade B) in Fulgoroidea was consistent: a more ancient Achilidae than Fulgoridae got strong supports, and the placement of these two families was congruent with previous studies based on either morphological or molecular data (Bourgoin, Steffen-Campbell & Campbell, 1997; Yeh, Yang & Hui, 2005; Urban & Cryan, 2007). Three families (Flatidae, Issidae and Ricaniidae) formed consistently a clade with strong nodal supports (BPP/BS = 1/100), in concordance with studies based on morphological, mitochondrial and nuclear genes (Muir, 1930; Asche, 1987; Urban & Cryan, 2007).

## Discussion

This study presents the description of five complete mitochondrial genomes of Achilidae species. Among these five mitogenomes, the A + T-rich region exhibited more variation in length, varying from 908 bp in *Pa*. sp. to 1,850 bp in *Pl*. sp., than other regions. Furthermore, as the length of control region decreases among taxa, so it does the number of repeat units and their copy numbers. This feature indicated that characteristics of these regions in Achilidae were taxon-specific, and the different sizes or copy numbers of repeat units had some influences on the size of the control regions and also the mitogenomes. Except for *nad4* and *nad5*, the length differences of each PCGs among five newly sequenced mitogenomes were no more than 20 bp, indicating relatively conserved features of protein-coding genes. The limited length variations in all tRNAs and rRNAs were also observed among different species, which was mainly due to their stable secondary structures.

The calculation of A + T content, AT-skew and GC-skew of 26 Fulgoroidea mitogenomes indicated a significantly biased toward A + T nucleotide, which was considered to be due to the abundance of adenines caused by the high ATP concentration (Xia, 1996), the high concentration of reactive oxygen species promoting the conversion from G:C to T:A or A:T, the low efficiency of the mitochondrial DNA repair system, and the requirement of maintaining translation efficiency (Kang & Hamasaki, 2002; Mason et al., 2003; Anastassopoulos, 2007).

In general, T was the most common nucleotide at second codon position (avg. 43%). The frequently usage of the fourfold degenerated codon NNA and the rarity of NNG in Fulgoroidea mitogenomes were also observed in other hemipterans (Hua et al., 2008; Liu et al., 2014; Song & Liang, 2009; Yuan et al., 2015), and may be associated with the nucleotide bias between AT and GC in mitogenomes (Hillis & Bull, 1993).

The length of the tRNAs is affected by the size of the TψC and the DHU arms which may have aberrant loop and very short stems varying in length within different tRNAs (Navajas et al., 2002). The anticodon arm was considered as the most conserved region of the tRNA, and each of the other three stems was always more conserved than the corresponding loop. Similarly, the conservation of three variable regions was also different (“extra” arm > the DHU arm > the TψC arm) (Navajas et al., 2002). The conservative secondary structures of tRNA genes appeared to accumulate much variation linearly for a long period of time, and had fewer restrictions on their tertiary structure than do nonmitochondrial tRNAs (Kumazawa & Nishida, 1993).

In both the ML and BI trees, the basal position of Delphacidae were supported with high probabilities. Placement of this family is consistent with previous hypotheses (Asche, 1987; Wilson et al., 1994), and is supported by the morphological (hind tarsal spines, ovipositor structure, and features of the adult female genitalia) (Muir, 1930; Asche, 1987; Emeljanov, 1990; Bourgoin, 1993) and molecular (18S, 16S and partial 28S rDNA sequences, Histone 3, and Wingless) phylogenies (Bourgoin, Steffen-Campbell & Campbell, 1997; Yeh & Yang, 1999; Yeh, Yang & Hui, 2005; Urban & Cryan, 2007). However, Chen & Yang’s (1995) phylogenetic tree, based on analysis of larval metatarsi, placed Delphacidae as a sister of Achilidae, suggesting a polarity reversal relative. Under both reconstruction methods, the Achilidae and Fulgoridae formed a sister clade with nodal supports 77 for ML and 0.99 for BI. The close relationship of these two families was also suggested by morphological data (Muir, 1930; Asche, 1987; Emeljanov, 1990; Bourgoin, 1993) and molecular results (Yeh, Yang & Hui, 2005; Urban & Cryan, 2007). Furthermore, the monophyly of Achilidae (Szwedo, 2004) has yet to be tested because of the limited number of available mitogenomes, and more detailed investigation is needed to test the monophyly of Achilidae. Placement of Ricaniidae sister to Flatidae and Issidae was consistently supported in both analyses with strong nodal supports. The sister relationship of these three families was also supported by the loss of the posterior tentorial arms observed in Flatidae and Ricaniidae (Emeljanov, 1990). Because of the limited mitogenome sequences, the current study did not support the paraphyly of Issidae, which was observed in other molecular phylogenetic analyses (Bourgoin, Steffen-Campbell & Campbell, 1997; Yeh, Yang & Hui, 2005).

The monophyly of a number of Fulgoroidea families recovered as monophyletic were supported by both morphological and molecular datas, but relatively limited taxonomic or biogeographic sampling could have contribution to the higher BS values, particularly for the families Achilidae or Delphacidae, which have greater taxonomic diversity in comparison to other planthopper families and occur in several biogeographic regions. The representative mitogenomes from Fulgoroidea are still limited, while phylogenetic analyses will be more reliable and convincing as more mitogenomes and genomes of the fulgoroidea species are available in databases.

The phylogenetic structure of 26 Fulgoroidea species supported in most previous studies and this study (Muir, 1930; Asche, 1987; Emeljanov, 1990; Bourgoin, 1993; Yeh, Yang & Hui, 2005; Urban & Cryan, 2007) might be related to their host plants and feeding locations. Wilson et al. (1994) suggested a greater association between more basal families and monocot hosts. Wilson et al. (1994) also proposed a greater association between the phylogenetic trend and feeding location: Achilidae species feed in underground or under bark, Delphacidae taxa feed near the ground, while members of the remaining families feed higher on their host plants. In this study, the structural and compositional analyses of Fulgoroidea mitogenomes were also partially congruent with the patterns of phylogenetic topologies. Hierarchical clustering of Fulgoroidea based on A + T content and AT-skew of whole mitogenomes, and nucleotide and amino acid compositions of the PCGs also divided Fulgoroidea into two main clades (Delphacidae and the other species). Furthermore, the gene arrangement of five tRNAs in Delphacidae mitogenomes was different from the gene order observed in Achilidae, Flatidae, Fulgoridae, Issidae and Ricaniidae mitogenomes. However, the internal relationships of Fulgoroidea excluding Delphacidae remained unclear, which might due to the minor base composition difference of mitogenomes among families.

Although the first five mitochondrial sequences of Achilidae were sequenced, it was still insufficient in comparison to a total of 161 genera and nearly 520 species in this family. Furthermore, only 26 mitogenomes downloaded from Genbank were included in the phylogenetic analyses. Considering the limited representatives of Fulgoroidea mitogenomes, a denser taxon sampling is still needed for further structural and compositional analyses and also for molecular classification of Fulgoroidea.

## Conclusions

In the present study, we firstly sequenced and analyzed the complete mitochondrial genomes of five Achilidae species (Hemiptera: Fulgoroidea). The five mitogenomes ranged from 15,214 to 16,216 bp in length, with the typical gene content and similar arrangement of genes usually observed in Hexapods. The 7-bp overlap “ATGATAA” between *atp8* and *atp6* was found in 26 Fulgoroidea species. Additionally, an 8-bp overlap “AAGCCTTA” between *trnW* and *trnC* was found for the first time in the mitogenomes of several Fulgoroidea taxa. In the 26 analyzed Fulgoroidea mitogenomes: the gene rearrangement of five tRNAs (*trnW*, *trnC* and *trnY*; *trnT* and *trnP*), the A + T content and AT-skew of the whole mitogenomes, and the nucleotide and amino acid compositions of the PCGs, revealed some family-level differences between family Delphacidae and the other five families (Achilidae, Flatidae, Fulgoridae, Issidae and Ricaniidae). The phylogenetic relationships constructed by both ML and BI methods were consistent and supported the monophyly of Fulgoroidea, a close affinity among the families Flatidae, Issidae and Ricaniidae, and a close relationship between Achilidae and Fulgoridae. We believe that the mitogenomes of the five Achilidae insects will be useful for further understanding of the evolutionary phylogenetic relationship within Fulgoroidea or even Hemiptera.

## Supplemental Information

10.7717/peerj.6659/supp-1 Supplemental Information 1 The mitogenome relative synonymous codon usage (RSCU) across 26 Fulgoroidea mitogenomes.

10.7717/peerj.6659/supp-2 Supplemental Information 2 Supplementary Tables 1–9.

## Additional Information and Declarations

### Data Availability

The following information was supplied regarding data availability: The sequence data of the five Achilidae mitogenomes are available at GenBank: MH324927–MH324931 for *Betatropis formosana*, *Magadhaideus luodiana* sp. nov, *Peltatavertexalis horizontalis* sp. nov, *Plectoderini* sp. and *Paracatonidia* sp., respectively.

### New Species Registration

The following information was supplied regarding the registration of a newly described species: Publication LSID: urn:lsid:zoobank.org:pub:D1E6581B-15A0-4277-B00E-0564ABD04DE1. Plectoderini sp. LSID: urn:lsid:zoobank.org:act:5A8F54C8-4EB7-4C4B-A8AA-9A2C497145EF, Magadhaideus luodiana sp. nov LSID: urn:lsid:zoobank.org:act:61F01D73-4279-4508-BD98-8F45BA1D7A50, Paracatonidia sp. LSID: urn:lsid:zoobank.org:act:CD9F8C3F-CF12-4AC6-8594-918EB19B68E1, Peltatavertexalis horizontalis sp. nov LSID: urn:lsid:zoobank.org:act:90B1FBF5-6BD7-480D-ABA4-B851035A456A.
