# Supplementary figures and images for: Comparative analysis of the complete mitochondrial genomes of five Achilidae species (Hemiptera: Fulgoroidea) and other Fulgoroidea reveals conserved mitochondrial genome organization
Source: PeerJ. 2019 Mar 26;7:e6659. doi: 10.7717/peerj.6659 (PMC6440461; doi:10.7717/peerj.6659)

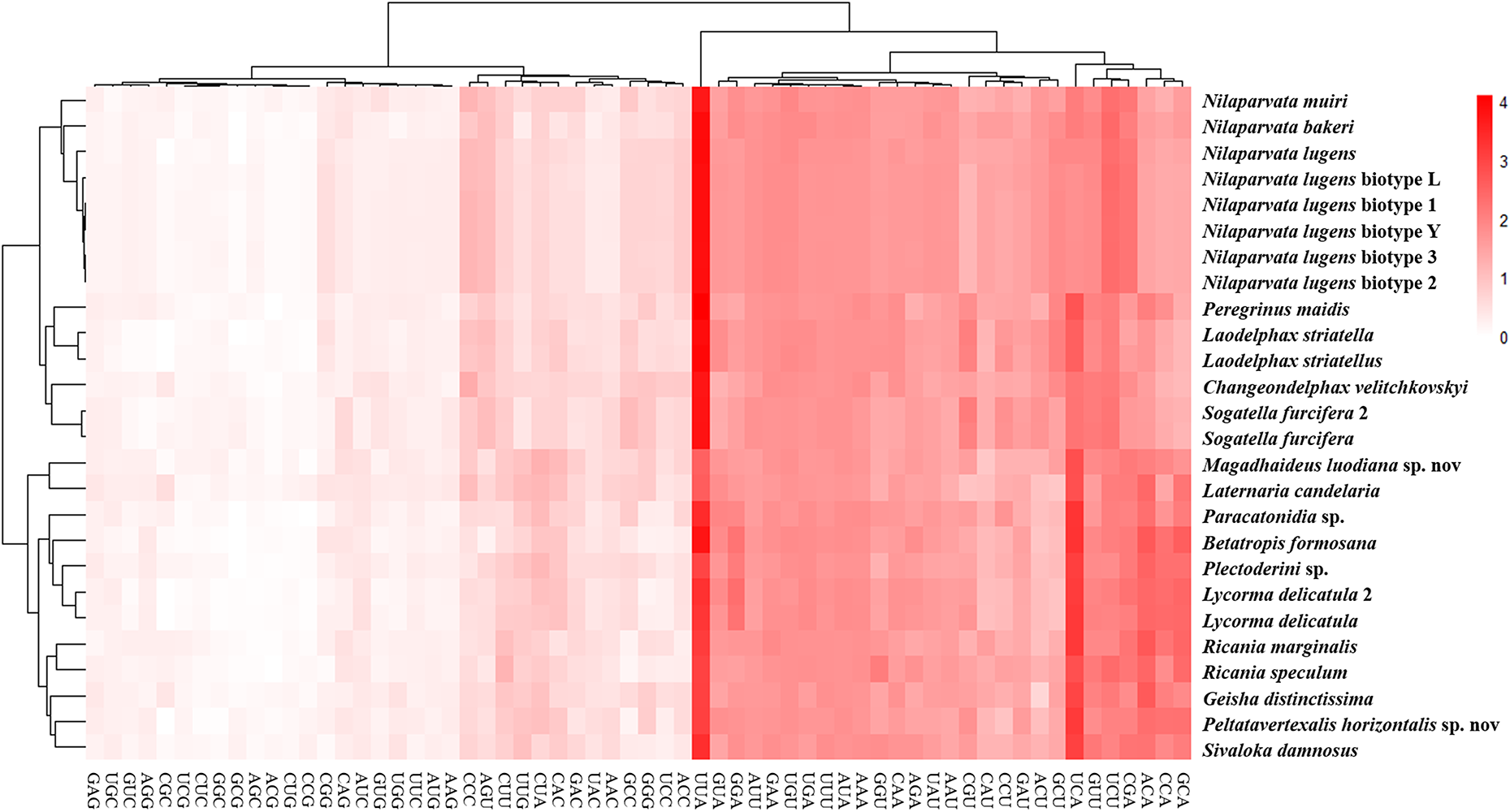

Supplement: Supplemental Information 1 [file peerj-07-6659-s001.png]
